# Enhancing the osteogenic potential of 3D-printed polycaprolactone/hydroxyapatite composite scaffolds via in-situ ZIF-8 surface modification

**DOI:** 10.1007/s10856-026-07029-y

**Published:** 2026-04-14

**Authors:** Saba Moslemi, Ghasem Dini, Fatemeh Ejeian, Aliakbar Najafinezhad, Sayede Tayebe Mousavi Mourkani

**Affiliations:** 1 Department of Nanotechnology, Faculty of Chemistry, University of Isfahan, Isfahan, 81746‑73441 Iran; 2 Department of Animal Biotechnology, Cell Science Research Center, Royan Institute for Biotechnology, ACECR, Isfahan, 81593-58686 Iran

## Abstract

Bone defects remain a major clinical challenge, necessitating advanced scaffolds that combine suitable mechanics, bioactivity, and osteoinductive cues for effective regeneration. This study developed 3D-printed polycaprolactone/hydroxyapatite (PCL/HA) composite scaffolds via fused deposition modeling and enhanced their surface with in-situ zeolitic imidazolate framework-8 (ZIF-8) modification to promote osteogenic performance. Hydrothermally synthesized HA nanoparticles exhibited high crystallinity, <100 nm size, and ~23 m²/g specific surface area. The optimal PCL + 25 wt.% HA composition achieved a compressive modulus of ~0.36 GPa and strength of ~17 MPa, within the range reported for human trabecular bone. The scaffolds demonstrated controlled biodegradation ( ~ 15% weight loss after 28 days in PBS) and strong bioactivity, with progressive apatite mineralization confirmed by SEM, XRD, and ion concentration changes in simulated body fluid over 28 days. ZIF-8 surface functionalization enabled sustained, non-burst Zn²⁺ release (0.18–1.66 ppm over 28 days) within safe biological limits. In vitro assays using MG-63 cells showed significantly improved cell adhesion, proliferation (MTS assay), and osteogenic differentiation on ZIF-8-modified scaffolds compared to unmodified controls, evidenced by 2.1-fold higher alkaline phosphatase (ALP) and 2.5-fold higher BMP2 gene expression after 21 days of induction. These results demonstrate that the synergistic combination of HA reinforcement and controlled Zn²⁺ release from ZIF-8 provides a multifunctional scaffold platform for bone regeneration.

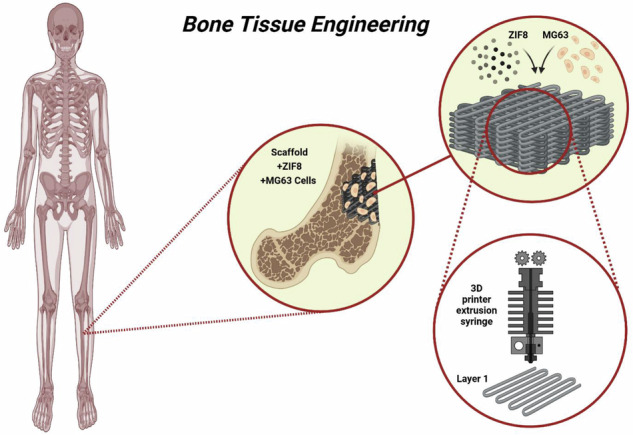

## Introduction

Bone is an essential connective tissue that provides structural support, enables movement, and stores vital minerals. However, bone defects—caused by trauma, fractures, surgeries, infections, or congenital disorders—pose significant medical challenges. With the aging global population, conditions such as osteoporosis and arthritis are becoming more prevalent, increasing the demand for effective bone repair solutions [1–3]. Although bone has a natural ability to heal, large or complex defects often require medical intervention. For centuries, bone grafting has been a primary approach to reconstruction. While effective, it has drawbacks, including infection risks, chronic pain, disease transmission, and poor mechanical performance. These limitations highlight the need for innovative biomaterials and engineered scaffolds to improve bone regeneration, offering safer and more efficient alternatives for patients [4–11].

Bone tissue engineering (BTE) presents a promising approach to repairing bone defects by integrating engineering principles with biological components. This strategy relies on scaffolds, bioactive agents, and cells to promote tissue regeneration. Scaffolds provide structural support, facilitate cell adhesion, and interact with the extracellular matrix to enhance bone healing. An ideal scaffold should be biocompatible, biodegradable, porous, and mechanically strong, while also supporting cell infiltration, vascularization, and nerve growth [12–19].

Material selection plays a critical role in scaffold design. While metals offer strength, their high stiffness and potential toxicity limit their biomedical applications. Ceramics, though biocompatible, are brittle and difficult to shape. Polymers and hydrogels alone lack sufficient mechanical properties. Since natural bone is a composite, combining polymers with ceramic nanoparticles achieves an optimal balance of strength, bioactivity, and biodegradability, making composite scaffolds the most viable option for bone regeneration [20, 21]. Bioceramics, widely used in bone composite scaffold fabrication, offer bioactivity, biocompatibility, bioresorption, and mechanical stability. In this study, hydroxyapatite (HA) is selected for its chemical similarity to bone, excellent osteoconductivity, and ability to promote osteoblast proliferation and stem cell differentiation. Its biocompatibility and controlled degradation make HA an ideal choice for enhancing bone regeneration and integration [22–25]. HA can be synthesized by methods like solid-state, hydrothermal, and chemical precipitation. This study uses the hydrothermal method, where high temperature and pressure produce highly crystalline nanoparticles, with temperature, reaction time, and pH affecting their final properties [26–29].

Polymers used in bone scaffolds are classified as natural or synthetic. Natural polymers like collagen, chitosan, and alginate lack the mechanical strength required for bone-like composites. In contrast, synthetic polymers—biodegradable and non-biodegradable—offer superior physicochemical properties and processability [30, 31]. This study utilizes polycaprolactone (PCL), an FDA-approved biodegradable polymer with suitable mechanical strength, flexibility, and thermal stability [20]. However, PCL has weak cell adhesion and a slow degradation rate, necessitating its combination with bioceramics like HA. Such composites enhance mechanical properties and bioactivity. Studies indicate that PCL/HA scaffolds improve compressive strength, cell proliferation, and mineralization. Cho et al. [32] demonstrated superior mechanical properties and apatite formation, while Krishna et al. [33] observed enhanced osteogenic differentiation, extracellular matrix deposition, and collagen formation. Combining PCL with HA addresses individual material weaknesses, making these composites highly promising for BTE.

Various techniques have been developed for fabricating bone composite scaffolds, including solvent casting, freeze-drying, gas foaming, and electrospinning [34]. An optimal scaffold production method should ensure reproducible structures with controlled porosity, as pore geometry significantly affects mechanical and biological responses. Three-dimensional (3D) printing is a particularly promising approach, allowing precise control over scaffold architecture [35]. This method constructs scaffolds layer by layer, improving structural uniformity and facilitating nutrient transport, bone ingrowth, and vascularization. 3D techniques such as stereolithography (SLA), selective laser sintering (SLS), and fused deposition modeling (FDM) are widely used in scaffold fabrication [34–36]. FDM enables cost-effective, precise, and mechanically suitable bone scaffolds, while 3D printing advances allow complex structures that mimic natural bone [37–39].

Surface modification of scaffolds using nanoparticles further improves their performance. Metal-organic frameworks (MOFs) have gained significant attention due to their nanoporous hybrid structures [40]. These materials exhibit thermal stability, very high surface area, low density, and excellent drug-loading capabilities. Among MOFs, zeolitic imidazolate frameworks (ZIFs) offer high porosity, stability, and biocompatibility [41–43]. ZIF-8, composed of Zn²⁺ ions and MIM-2 ligands, provides pH responsiveness, controlled degradability, and bone-inducing properties [44]. To enhance MOF adhesion, scaffold surfaces undergo thermal oxidation or polydopamine (PDA) coating [45]. Various synthesis methods exist, including sonochemical, mechanochemical, sol-gel, and microwave techniques, but the in-situ method is the most efficient, promoting rapid nanoparticle growth. Zhou et al. [46] demonstrated that scaffolds containing ZIF-8 nanoparticles exhibited improved mechanical properties, enhanced cell adhesion and proliferation, increased apatite formation, superior osteoinductivity, and strong antibacterial characteristics. These advantages make MOF-functionalized scaffolds promising candidates for BTE applications.

**Scheme 1:**
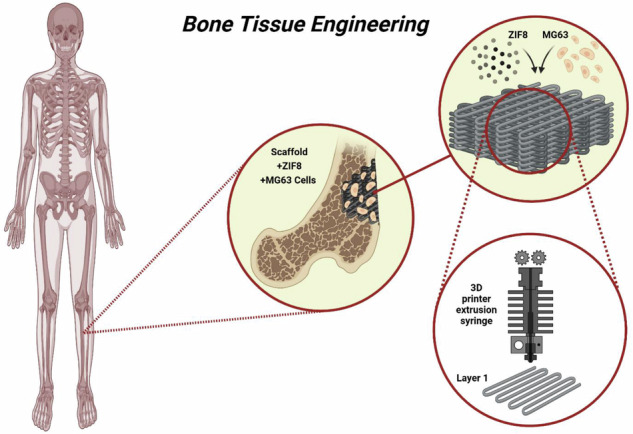
A graphical illustration of a 3D-printed PCL/HA bone tissue engineering scaffold surface modified with ZIF-8 MOFs

While PCL/HA composites offer mechanical strength and biocompatibility, they often lack the bioactivity needed for optimal performance. To tackle these issues, this study presents a novel approach: a 3D-printed PCL/HA scaffold enhanced with in-situ ZIF-8 surface modification. This innovative design stands out by combining ZIF-8’s pH-responsive properties and controlled Zn²⁺ release to boost osteogenic differentiation while maintaining structural integrity and enhancing cytocompatibility. Unlike prior studies that relied solely on HA reinforcement or alternative surface treatments, our method integrates these elements synergistically. HA nanoparticles, synthesized hydrothermally with high crystallinity and a mesoporous structure, improve osteoconductivity, and the optimized 25 wt.% HA composition delivers mechanical properties closely aligned with trabecular bone. Through detailed characterization—including compressive strength, bioactivity in simulated body fluid (SBF), and in vitro cellular responses—we demonstrate the scaffold’s strong potential for bone regeneration, as illustrated in Scheme 1

## Materials and methods

### Materials

For HA nanoparticle synthesis, Calcium Carbonate (CaCO₃, Merck), Phosphoric Acid (H₃PO₄), distilled water, and Ammonia (NH₃, Merck) were utilized. Composite fabrication for 3D printing involved PCL (Mw = 80 kDa, Sigma-Aldrich) and Chloroform (CHCl₃, Merck). For the in-situ synthesis of ZIF-8 nanoparticles, Polyethylenimine (Sigma), Tris buffer (Merck), Dopamine hydrochloride (Sigma), Zinc Nitrate Hexahydrate (Zn(NO₃)₂·6H₂O, Sigma), and 2-Methylimidazole (Sigma) were used.

Biological assessments employed Phosphate-Buffered Saline (PBS), Simulated Body Fluid (SBF), MG-63 cell line (Pasteur Institute of Iran), Fetal Bovine Serum (FBS, GIBCO), DMEM, Glutamax (GIBCO), ethanol, Penicillin–Streptomycin, Trypsin-EDTA (GIBCO), MTS assay reagent (Promega), TRITC Phalloidin, and DAPI (Sigma-Aldrich).

### Synthesis of HA powder

HA powder was synthesized using a hydrothermal method. First, 5 g of CaCO₃ was mixed with 4000 μL of 85% H₃PO₄ dissolved in 75 mL of distilled water and stirred for 30 min. After adding CaCO₃, the mixture was stirred for an additional 1 h. To enhance solubility, the solution underwent ultrasonic treatment for 20 min. The pH was adjusted to 7–7.5 using 25% ammonia, ensuring the prevention of unwanted phase formation. The solution was then sealed in an autoclave and heated to 180 °C for 40 h. After synthesis, the sediment was washed multiple times to remove salts. Finally, the wet powder was dried at 80 °C for 24 h and ground using a mortar for further processing.

### Characterization of HA powder

The crystalline structure and phase composition of HA powder were analyzed using X-ray diffraction (XRD) (Bruker D8 Advance, Germany) with Cu_Kα radiation (λ = 1.54 Å). The morphological characteristics were examined via field-emission scanning electron microscopy (FE-SEM, ZEISS SIGMA VP-500). Porosity and active surface area were assessed using Brunauer-Emmett-Teller (BET) analysis. Accordingly, the textural properties of HA powder, including specific surface area, pore volume, and average pore diameter, were determined through nitrogen gas adsorption/desorption at liquid nitrogen temperature ( ~ 77 K) using a BEL SORP mini II series instrument.

### Fabrication of PCL/HA composites

To fabricate composite materials, HA powder was sieved and dispersed in chloroform. Composites containing 20, 25, and 30 wt.% HA were prepared by mixing appropriate amounts of HA with PCL to obtain at least 2 g of feed for the 3D printer syringe. PCL was dissolved in chloroform to form a clear solution, into which HA powder was gradually added and thoroughly mixed, resulting in a milky, creamy composite. This mixture was stirred for 24 h to ensure uniform suspension while allowing chloroform to evaporate, forming a solid composite that could be easily removed from the container. After separation, the composite was cut into small pieces and loaded into the 3D printer nozzle. The selected HA concentrations (20, 25, and 30 wt.%) were determined through preliminary optimization, during which HA loading was progressively increased to identify the maximum printable composition permitted by the extrusion-based 3D printing system (Section 2.5). While higher HA contents were investigated, formulations exceeding 30 wt.% showed inadequate rheological behavior and unstable extrusion, preventing continuous filament formation. Therefore, compositions near this threshold were selected for systematic mechanical evaluation.

### 3D printing of PCL/HA scaffolds

This study employed the 3DPL Bioprinter N1 (3DPL®, Iran) operating in FDM mode to fabricate composite scaffolds. Key printing parameters included a layer height of 0.20 mm, infill density of 40% (rectilinear pattern), default print speed of 70 mm/min, bed temperature of 25 °C, and nozzle diameter of 0.50 mm. G-code files were generated using the 3DPL-Software with standard post-processing. Pore size and strand distance were defined by the scaffold CAD model geometry, typically featuring 0.5 mm strand spacing in a 0°/90° grid pattern for porous constructs. The composite materials (containing 20, 25, and 30 wt.% HA powder) were loaded into a cylindrical syringe, with a compressive tool applied to ensure uniformity and eliminate voids for consistent flow. Extrusion was performed through a 0.5 mm nozzle at approximately 140 °C extruder temperature and 3 bar pneumatic pressure, controlled by pre-programmed G-code for precise deposition onto the platform. This approach enabled successful fabrication and subsequent analysis of the HA-reinforced scaffolds.

### Characterization of the fabricated scaffolds

Scaffolds with three different HA percentages were subjected to compression testing using a 2 T SANTAM device. Samples (*n* = 5) measuring approximately 1 × 1 × 0.5 cm³ were selected, and all dimensions were recorded. The applied pressure was gradually increased until reaching 70% of the sample’s height, at which point the test was halted. The compression speed was set to 0.2 mm/s. Based on the mechanical test results, the optimal scaffold for future studies was identified as the one containing 25 wt.% HA, which exhibited superior mechanical properties. The detailed results of these tests are presented in the Results section.

The bioactivity of the optimized scaffold was assessed by immersing it in SBF. The scaffold was cut into ~ 5 × 5 × 5 mm³ (*n* = 3), washed with 70% ethanol, and sterilized under UV light for 25 min. The sterilized samples were then incubated in 10 mL of SBF solution at 37 °C for 28 days [47]. To evaluate apatite formation on the scaffold surfaces, scanning electron microscopy (SEM, Philips XL30) and the XRD method were used. Additionally, to understand the bioactivity mechanism, the pH of the SBF solution containing the scaffolds was monitored, and changes over time were analyzed. Finally, inductively coupled plasma atomic emission spectroscopy (ICP-OES, Analytik Jena PQ 9000 instrument) was used to measure the concentration of ions released from the samples after immersion in SBF for up to 28 days.

A degradation test was conducted to evaluate the behavior of the optimized PCL/HA scaffold. Samples ( ~ 5 × 5 × 5 mm³, *n* = 3) were weighed before immersion in PBS and sterilized using UV light and ethanol. The samples were then incubated at 37 °C for 1, 3, 5, 7, 14, 21, and 28 days. After incubation, the samples were removed from PBS, placed on filter paper, and dried in a vacuum oven at 40 °C for 48 h to ensure complete moisture removal. Once dried, all samples were reweighed to determine weight loss. The pH of the PBS solution was also monitored at specific time intervals to assess degradation kinetics. Finally, the weight loss of the scaffolds was calculated using Eq. 1 as follows:

1 $${Deg}r{adation}\,( \% )=\frac{({w}_{0}-{w}_{t})}{{w}_{0}}\times 100$$

where *w₀* represents the initial weight and *w*_*t*_ is the final weight after immersion and drying.

### Synthesis of ZIF-8 nanoparticles

#### Dopamine coating on the PCL/HA scaffold

To ensure cleanliness before coating, PCL + 25 wt.% HA scaffold samples (1 × 1 × 0.5 cm³) were immersed in pure ethanol for 30 min. For dopamine coating, a DA/PEI solution (2 mg/mL each) was prepared in Tris buffer (10 mM, pH 8.5). The correct order of addition—PEI first, followed by dopamine—was confirmed by the appearance of a yellowish tint in the solution. The samples were then immersed in the prepared solution and gently shaken for 5 h, allowing sufficient aeration for dopamine precipitation and a visible color change. Finally, the coated samples were washed with distilled water and dried at 37 °C for 24 h [44].

#### In-situ synthesis of ZIF-8 on the dopamine-coated scaffold surface

Each Falcon tube contained 1.32 g of 2-methylimidazole and 0.0548 g of zinc nitrate, both dissolved in 5 mL of distilled water. The dried PDA-coated scaffolds were placed in containers, followed by the addition of the zinc nitrate solution, allowing 1 min for zinc ion deposition on the scaffold surface. The 2-methylimidazole solution was then added, leading to the formation of white, milky particles, indicating successful ZIF-8 nanoparticle synthesis. The scaffolds were incubated at room temperature for 2.5 h to facilitate in-situ crystallization of ZIF-8 nanoparticles, without aeration or agitation. After incubation, the samples were washed and dried, following the same procedure as in the dopamine coating step [44]. To confirm nanoparticle formation, FE-SEM analysis equipped with energy-dispersive X-ray spectroscopy (EDS) was performed. Finally, the samples were divided into two groups for biological assessment: one without ZIF-8 (labeled Scaffold1) and one with ZIF-8 (labeled Scaffold2).

### In vitro biological assays

#### Cell culture

The MG-63 osteoblastic cell line was used to evaluate the optimized scaffolds. Cells were cultured in DMEM supplemented with 10% FBS, 1% GlutaMAX, 1% penicillin/streptomycin, and 1% essential amino acids, ensuring that all solutions were pre-warmed to body temperature. Samples (Scaffold1 and Scaffold2) were sterilized by immersion in 70% ethanol for 48 h, rinsed with sterile distilled water, dried, and further sterilized using a UVC sanitizer box. To maintain sterility, containers were sealed with Parafilm.

To create a hydrophobic surface for cell adhesion, agar plates were prepared using a 1.5% agar solution, autoclaved, and poured into wells. Sterilized scaffolds were then placed in 24-well plates for cell seeding. Once the cells had proliferated in the incubator and reached a minimum density of 90%, 5 × 10³ cells were seeded onto each sample. After initial cell attachment, an additional complete medium was added, and the plates were incubated. The cells were allowed to adhere for 4 h before adding more medium for further culture.

#### Cell adhesion

To assess cell functionality and health, fluorescent staining of nuclei and actin filaments was performed using DAPI and phalloidin. Phalloidin binds to actin filaments, while DAPI binds to DNA, enabling visualization of both live and fixed cells. Samples with cultured cells were fixed on day 3, following a protocol that included washing with PBS, fixing with 4% paraformaldehyde for 30 min, permeabilization with 0.1% Triton X-100 for 30 min, and blocking with 5% FBS for 30 min to enhance dye absorption. The samples were then stained with phalloidin and DAPI, and marker expression was analyzed using fluorescence microscopy.

#### Viability and proliferation assays

Cell viability on the samples was evaluated using an MTS assay, which measures mitochondrial enzyme activity, converting MTS into a colored soluble salt to indicate viable cells. Measurements were taken on days 1, 3, 5, and 7. To perform the assay, the upper medium was removed from the samples, and a mixture of 400 µL of complete medium and 40 µL of MTS solution was added to each well. Plates were incubated in the dark for 3.5 h to prevent interference in absorbance readings. For accuracy, both a control group with cells and a cell-free control were included. Absorbance was measured at 492 nm using an ELISA reader, with net absorbance calculated by subtracting the control sample values from the test sample values.

#### Cell differentiation

The osteoinductive potential of the samples was assessed after a 21-day induction period under osteogenic conditions. Once the cells adhered to the samples, the growth medium was replaced with an osteogenic medium containing 50 mg/mL ascorbic acid, 10 mM β-glycerophosphate, and 10 nM dexamethasone. To facilitate osteogenic differentiation, the medium was renewed every three days. After 21 days, the cells were collected using the TRI reagent for RNA extraction, which was then used to synthesize complementary DNA (cDNA). The expression levels of alkaline phosphatase (ALP) and bone morphogenetic protein 2 (BMP2)—markers of early and late osteoblast differentiation, respectively—were analyzed via real-time PCR using SYBR Green detection.

### Statistical analysis

All quantitative experiments were conducted in at least three replicates, and results are presented as mean ± standard deviation (SD). For PCR analysis, statistical comparisons among groups were performed using one-way analysis of variance (ANOVA) followed by Tukey’s post-hoc test. A significance level of *P* < 0.05 was considered statistically significant. Statistical analyses were carried out using GraphPad Prism software.

## Results

### Characterization of synthesized HA powder

**Fig. 1:**
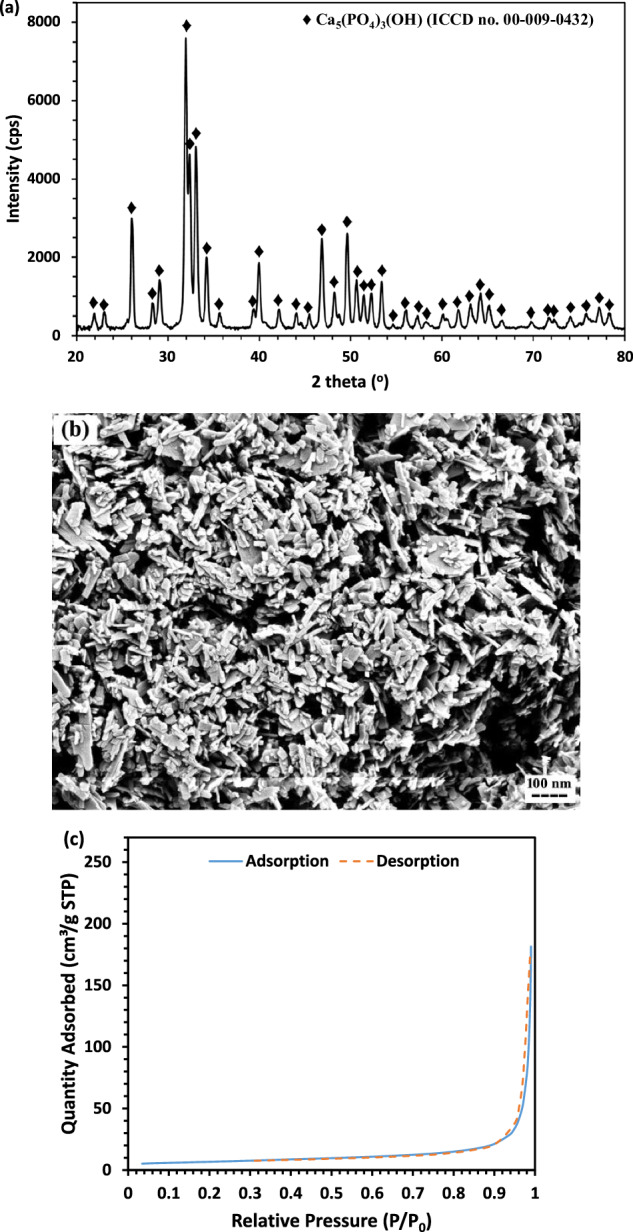
**a** XRD pattern, **b** SEM image, and **c** nitrogen adsorption/desorption isotherms at 77 K for the hydrothermally synthesized HA powder in this study

The phase composition of the hydrothermally synthesized HA powder was analyzed by XRD (Fig. 1a). The diffraction pattern exhibited sharp and well-defined peaks corresponding to highly crystalline, single-phase HA. No diffraction peaks related to CaCO₃ or CaO were detected. The morphology of HA powder was examined by SEM (Fig. 1b). The particles exhibited predominantly flaky and elongated structures, with sizes generally below 100 nm. In addition, BET analysis was performed to evaluate the textural properties of the synthesized HA nanopowders, including specific surface area and pore diameter. The nitrogen adsorption/desorption isotherms at 77 K (Fig. 1c) indicated a porous and relatively uniform structure. The specific surface area was measured to be ~ 23 m²/g, and the average pore diameter was approximately 15 nm.

### Characterization of the fabricated composite scaffolds

**Fig. 2:**
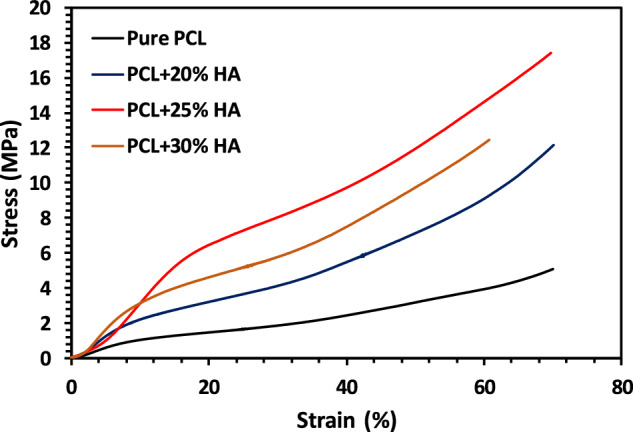
Compressive stress-strain curves of the 3D-printed pure scaffold and scaffolds containing three different percentages of HA nanoparticles

The mechanical performance of the three different composite scaffolds fabricated in this study was evaluated, and the resulting compressive stress-strain curves are presented in Fig. 2. As strain increased, the curves deviated from linearity, showing variations in strength and failure strain depending on HA content. According to Table 1, increasing HA content up to 30 wt.% resulted in compressive modulus and strength values of 0.43 ± 0.04 GPa and 16.7 ± 0.4 MPa, respectively. In comparison, the pure 3D-printed PCL scaffold exhibited a modulus of 0.14 ± 0.04 GPa and a strength of 5.1 ± 0.3 MPa. Table 1 shows that the scaffold containing 25 wt.% HA exhibited the highest yield strength and compressive strength.

**Table 1:** Mechanical properties of the 3D-printed pure scaffold and scaffolds with three different percentages of HA nanoparticles

Composition	Compressive modulus (GPa)	Yield strength (MPa)	Compressive strength (MPa)
Pure PCL	0.14 ± 0.04	1.1 ± 0.2	5.1 ± 0.3
PCL + 20 wt.% HA	0.27 ± 0.03	1.9 ± 0.3	12.2 ± 0.3
PCL + 25 wt.% HA	0.36 ± 0.05	5.6 ± 0.3	17.4 ± 0.2
PCL + 30 wt.% HA	0.43 ± 0.04	2.4 ± 0.1	16.7 ± 0.4

**Fig. 3:**
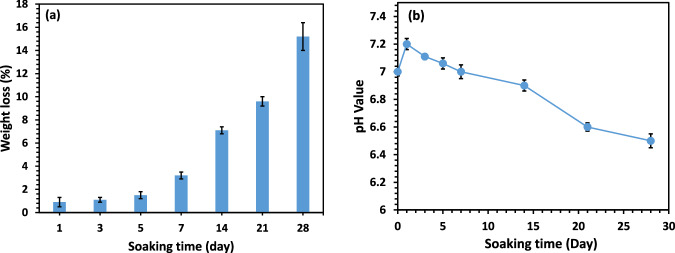
**a** Weight loss of scaffolds and **b** changes in the pH level of the PBS solution during 28 days of immersion at 37 °C

The weight loss of the optimal scaffold (PCL + 25 wt.% HA) was monitored over 1, 3, 5, 7, 14, 21, and 28 days of immersion in PBS (Fig. 3a). Weight loss remained relatively stable during the first seven days, with a notable increase beginning on day 14, reaching approximately 15% by day 28. Pure PCL scaffolds showed no significant weight loss during this period. In addition, the pH of the PBS solution during scaffold immersion was also monitored (Fig. 3b). The pH initially increased from approximately 7 to 7.2 on day 1, followed by a gradual decrease over the subsequent days.

**Fig. 4:**
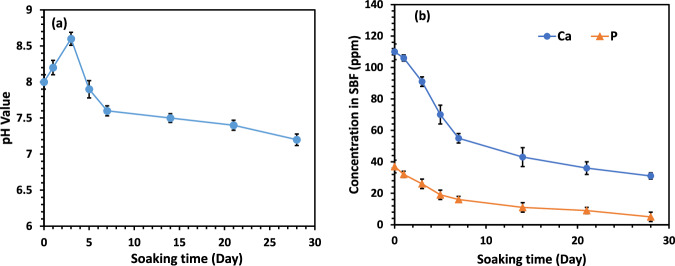
**a** Changes in pH and **b** changes in the concentrations of calcium and phosphorus ions in the SBF solution during a 28-day immersion at 37 °C

Figure 4a illustrates the pH changes in the SBF solution containing the optimized scaffolds over a 28-day immersion period. During the first three days, the pH increased slightly. After day 3, the pH gradually declined. Moreover, ICP-OES analysis was conducted to measure calcium and phosphorus ion concentrations in SBF during the 28-day immersion, with results shown in Fig. 4b. A gradual reduction in both calcium and phosphorus ion concentrations was observed starting from day 1 and continued throughout the immersion period.

**Fig. 5:**
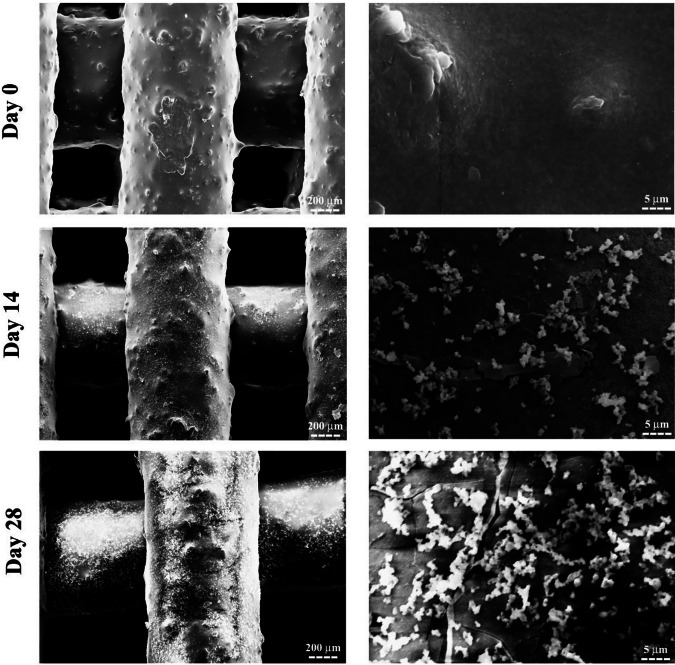
SEM images of the 3D-printed PCL + 25 wt.% HA scaffold showing mineralization after immersion in SBF solution for 0, 14, and 28 days

The SEM images illustrating the surface morphology of the 3D-printed PCL + 25 wt.% HA scaffold before and after immersion in SBF solution are presented in Fig. 5. After 14 days of immersion, an apatite layer began forming on the scaffold surface, as evident in the SEM images. SEM images on day 28 confirm that prolonged immersion in SBF led to the formation of a dense layer of fine apatite particles. The optimized scaffolds exhibited clear bioactive properties, making them suitable substrates for apatite formation and BTE applications.

**Fig. 6:**
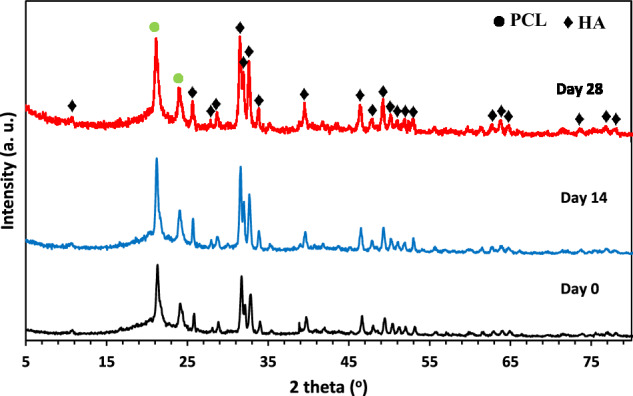
XRD patterns of the 3D-printed PCL + 25 wt.% HA scaffold after 0, 14, and 28 days of immersion in SBF solution

Figure 6 presents the XRD patterns of the 3D-printed PCL + 25 wt.% HA scaffold after 14 and 28 days of immersion in SBF. Peaks corresponding to the apatite structure became evident in the PCL/HA composite scaffold after 2 and 4 weeks, indicating the interaction of calcium and phosphate ions with the scaffold surface. The penetration of these ions into the scaffold’s porous structure further promoted apatite deposition. After 28 days of immersion, the intensity of the HA peaks increased, reflecting a greater degree of apatite crystallinity.

As stated before, the HA content in the PCL/HA scaffold was optimized by evaluating composites with 20, 25, and 30 wt.% HA, with mechanical testing identifying 25 wt.% as the ideal balance closely aligned with trabecular bone properties (Table 1). For the ZIF-8 coating, concentrations of zinc nitrate and 2-methylimidazole were selected based on established protocols [40, 44], ensuring consistent nanoparticle formation while preserving scaffold porosity and structural stability. This study prioritized establishing the combined efficacy of 25 wt.% HA and ZIF-8 functionalization, with their synergy validated by enhanced osteogenic performance (Section 3.3).

**Fig. 7:**
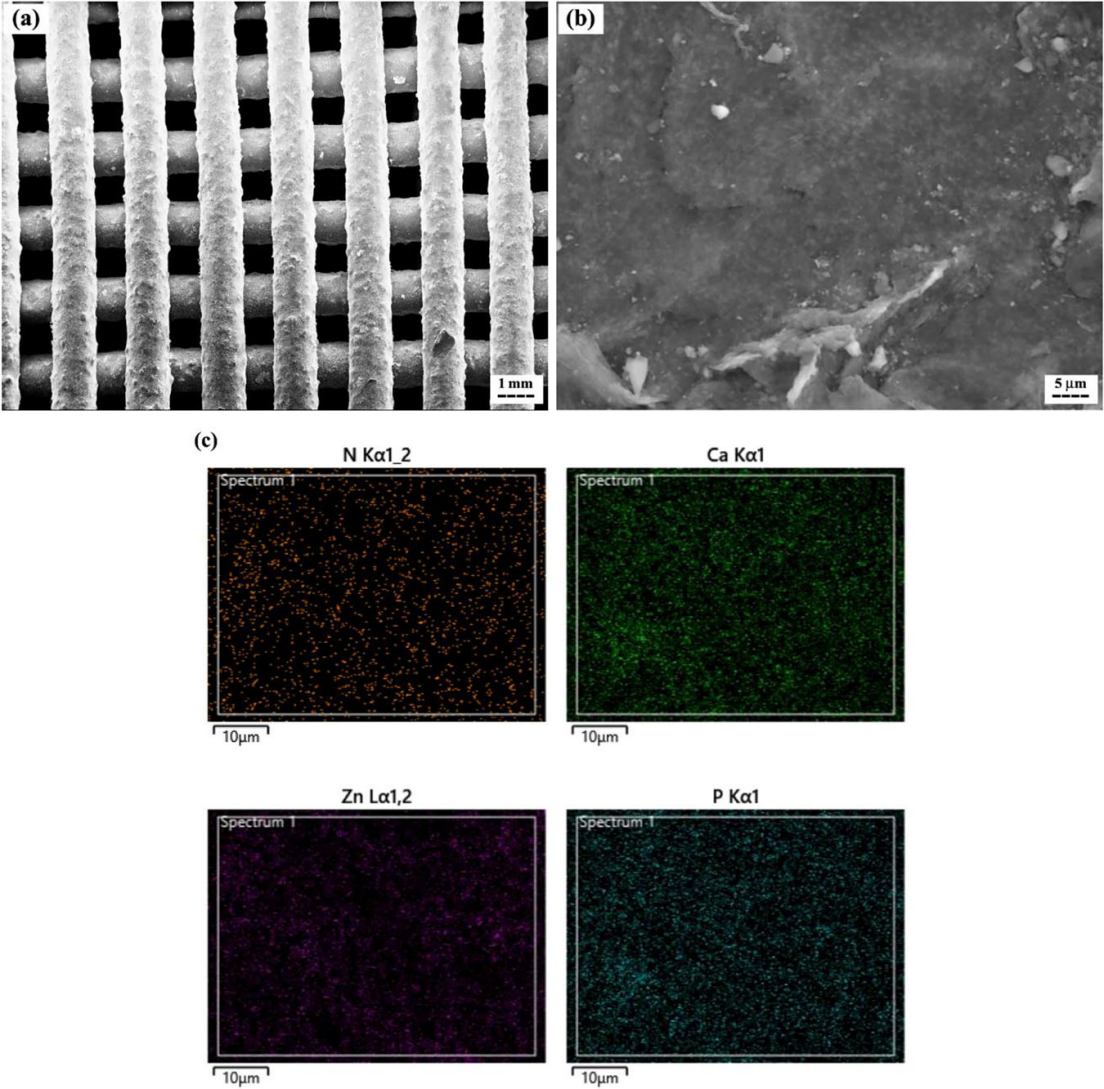
**a, b** SEM images of the 3D-printed PCL + 25 wt.% HA scaffold without surface modification (Scaffold1) at two different magnifications, and **c** corresponding EDS mapping

Figure 7 presents the EDS elemental mapping analysis and SEM images at different magnifications for the 3D-printed PCL + 25 wt.% HA scaffold without ZIF-8 surface modification (Scaffold1).

The SEM image (Fig. 7a) reveals a uniform three-dimensional porous structure, with pore sizes of approximately 350 µm, smaller than the designed strand spacing (0.5 mm), primarily due to material spreading upon deposition and partial strut fusion between layers. At higher magnifications (Fig. 7b), HA nanoparticles were observed to be evenly distributed within the PCL matrix. Figure 7c presents the elemental mapping analysis, confirming the presence of calcium and phosphate, indicating successful incorporation of HA nanoparticles within the PCL matrix.

**Fig. 8:**
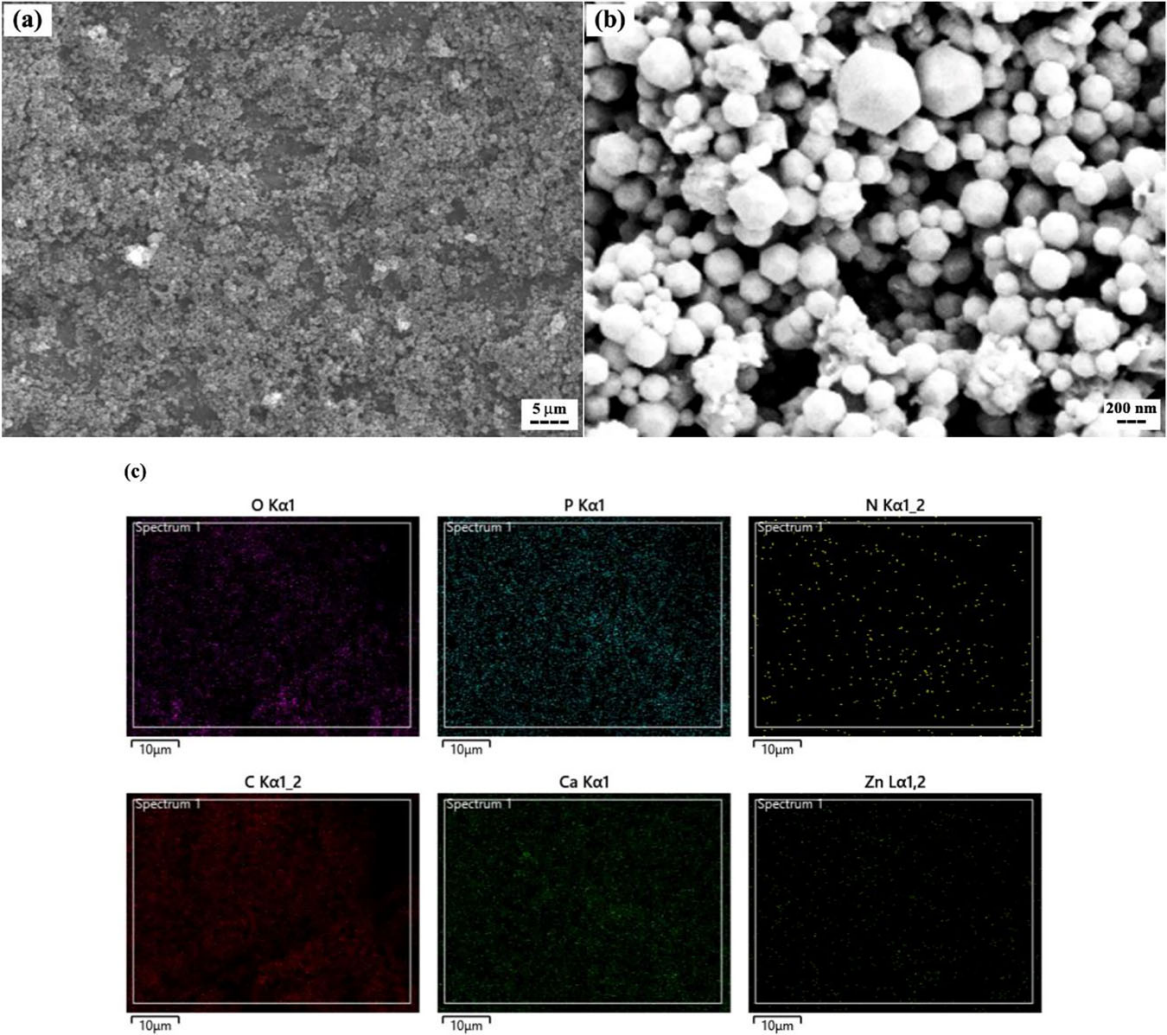
**a, b** SEM images of the optimized 3D-printed PCL + 25 wt.% HA scaffold after surface modification with ZIF-8 nanoparticles (i.e., Scaffold2), and **c** corresponding EDS mapping

SEM images (Fig. 8a and b) confirmed the formation of the ZIF-8 layer on the scaffold surface (Scaffold2). The observed particle sizes of ZIF-8 ranged from 50 to 300 nm. Figure 8c presents the elemental composition of the ZIF-8 layer. The presence of carbon, oxygen, nitrogen, and zinc confirms the formation of this MOF. Minor traces of calcium and phosphorus were also detected, originating from the HA substrate beneath the ZIF-8 layer.

**Fig. 9:**
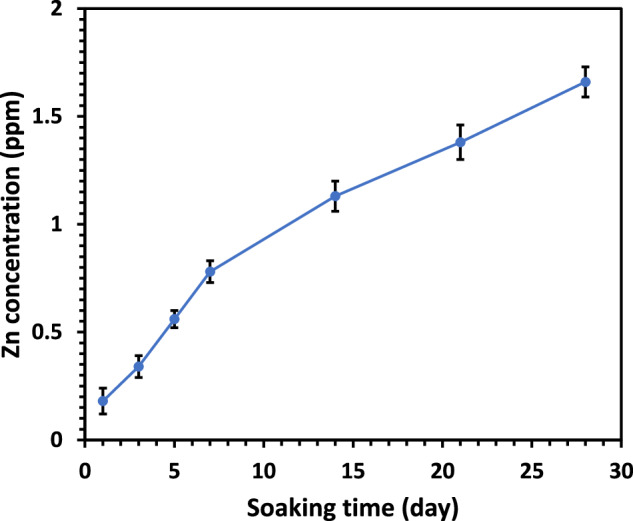
Release behavior of Zn²⁺ ions from the ZIF-8–modified PCL/25 wt.% HA composite scaffold in PBS over 28 days

The release kinetics of Zn²⁺ ions from ZIF-8–surface-functionalized PCL/HA composite scaffolds were investigated in PBS over 28 days (Fig. 9). Zinc concentrations gradually increased from 0.18 ppm on day 1 to 1.66 ppm by day 28, showing a controlled and sustained release profile. No initial burst release was observed, indicating the structural integrity of the ZIF-8 coating and stable encapsulation of nanoparticles within the PCL/HA matrix. Throughout the 28-day evaluation period, the release of Zn²⁺ ions remained gradual, with a peak concentration of 1.66 ppm, which is within the biologically safe range.

### In vitro biological assays

To systematically evaluate the biological performance of the fabricated scaffolds, a series of in vitro assays was conducted using MG-63 osteoblast-like cells. Cell adhesion was assessed through fluorescent staining of nuclei (DAPI) and actin filaments (Phalloidin) on day 3. Cell viability and proliferation were monitored via MTS assays over 1, 3, 5, and 7 days. To investigate osteogenic potential, real-time PCR analysis measured the expression of key markers after 21 days of osteogenic induction. These assays were designed to cover critical aspects of cellular response, from initial adhesion to long-term differentiation, providing a comprehensive evaluation of scaffold bioactivity. While additional time points or assays could further enrich the dataset, the current findings establish a strong foundation for the scaffolds’ osteogenic capabilities, with future studies planned to explore extended culture periods.

**Fig. 10:**
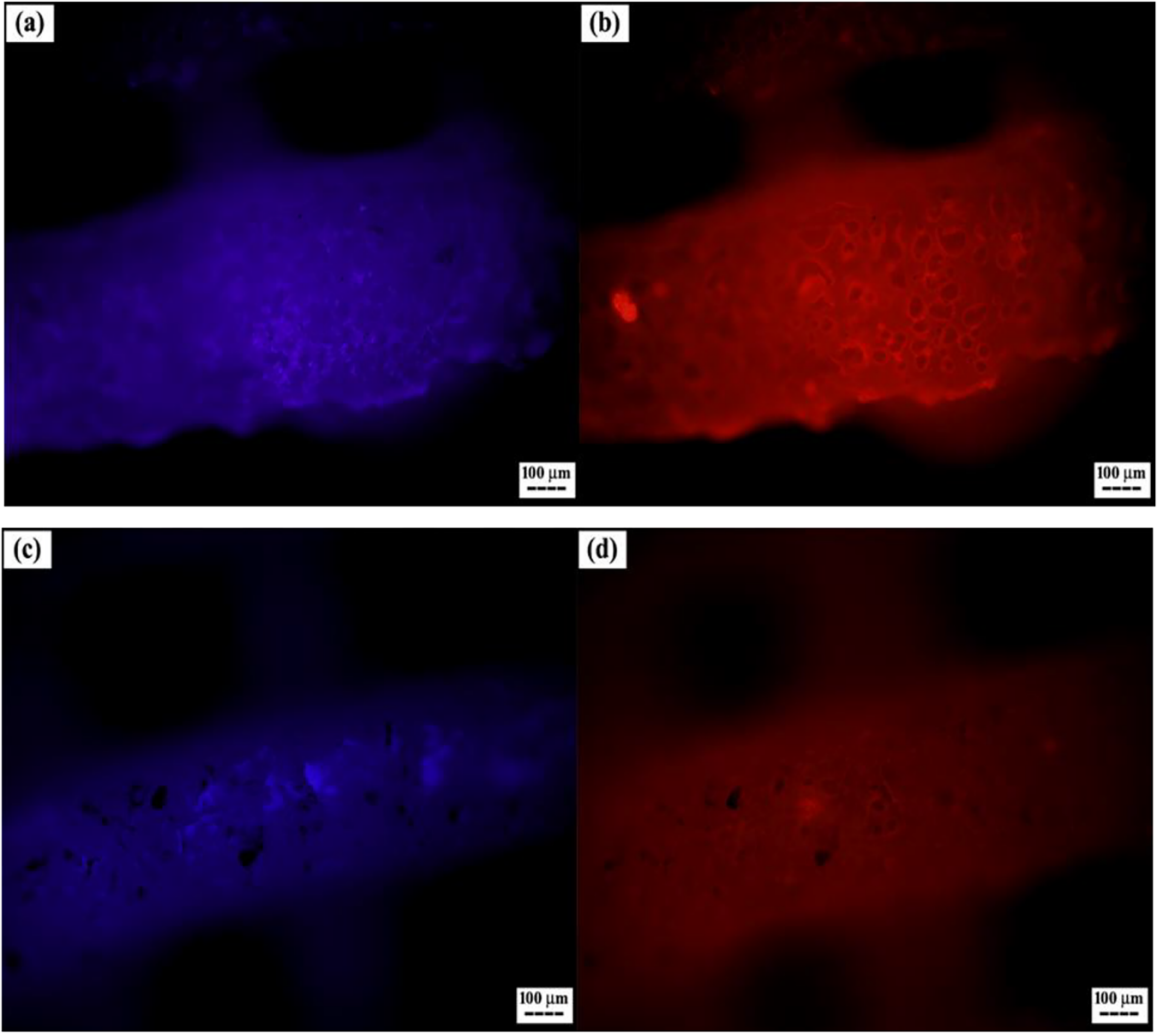
Fluorescent staining of cells on scaffolds: **a, c** Cell nuclei on Scaffold1 and Scaffold2 on day 3, respectively, and **b, d** Actin filaments in cells on Scaffold1 and Scaffold2 on day 3, respectively (Blue = DAPI, Red = Phalloidin)

**Fig. 11:**
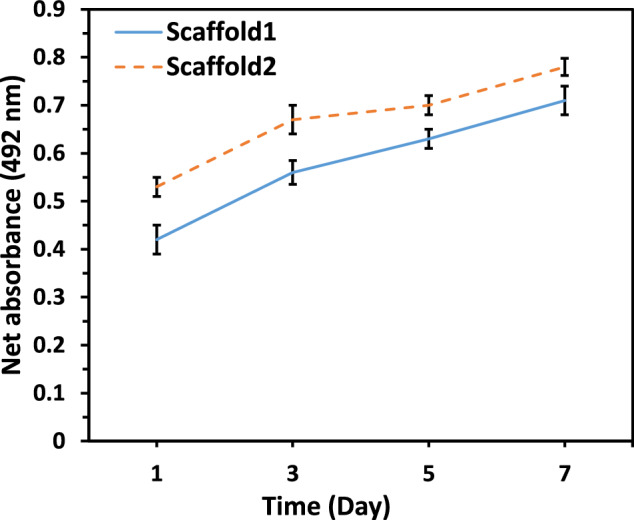
Cell viability results (MTS assay) for Scaffold1 and Scaffold2 over time, normalized to the absorbance of a cell-free MTS solution. Data are presented as mean ± SD from three independent experiments

**Fig. 12:**
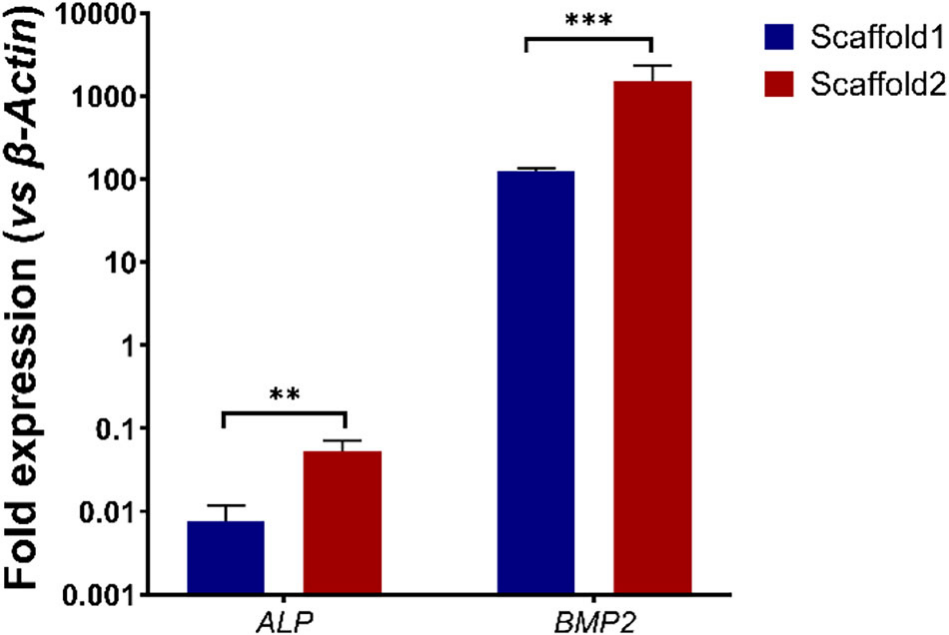
Real-time PCR analysis after three weeks of osteoinduction, measuring the relative expression of ALP and BMP2 compared to pre-treated cells (day 0). Data are presented as mean ± SD (*n* = 3). Statistical analysis was performed using one-way ANOVA followed by Tukey’s post-hoc test. **P < 0.01; ***P < 0.001

The DAPI-stained images in Fig. 10a and c depict Scaffold1 and Scaffold2 after 3 days of cell culture. Phalloidin-stained images in Fig. 10b and d reveal cytoskeletal organization and cytoplasm distribution in cells seeded on both scaffold types. DAPI staining of Scaffold1 (Fig. 10a) shows strong cell adhesion, with a relatively high cell density on the printed scaffold fibers. Well-defined nuclei and cytoplasm indicate successful attachment. By day three, cells also exhibited acceptable adhesion on Scaffold2. Nuclear clustering observed in Fig. 10c suggests an even greater number of cells attached to Scaffold2, likely due to enhanced surface interactions. Overall, the staining results confirm robust cell adhesion and appropriate cellular activity on both scaffolds after 3 days. Complementary assays, including MTS testing over 7 days (Fig. 11) and osteogenic marker expression after 21 days (Fig. 12), demonstrate prolonged cell viability and differentiation.

The MTS assay was conducted to assess the viability of MG-63 cells cultured on both scaffolds at 1, 3, 5, and 7 days (Fig. 11). The assay confirmed that MG-63 cells successfully adhered to both Scaffold1 and Scaffold2 on the first day, demonstrating initial cell survival. The increasing trend in Formazan absorption up to day 7 indicates enhanced cell viability, likely due to cell proliferation and expansion on the scaffold fibers. The growth curve in the logarithmic phase shows that the cells maintained their ability to increase in density, suggesting that proliferation may continue beyond 7 days. Scaffold2 exhibited higher absorbance values than Scaffold1, indicating greater cell adhesion, proliferation, and survival rates. Overall, the results demonstrate that both scaffolds were non-toxic and biocompatible, with no observed cytotoxic effects. Cells remained viable, showed no abnormalities, and did not undergo lysis upon contact with the scaffold materials.

Real-time PCR analysis (Fig. 12) demonstrated that ZIF-8 functionalization significantly enhanced osteogenic differentiation of MG-63 cells. ALP expression was upregulated by 2.1-fold and BMP2 expression by 2.5-fold in ZIF-8-coated scaffolds (Scaffold2) compared to uncoated scaffolds (Scaffold1). Statistical analysis using one-way ANOVA followed by Tukey’s post-hoc test revealed significant differences: ALP (*P* < 0.01) and BMP2 (*P* < 0.001). The results indicate enhanced early-stage osteoblastic activity (ALP) and improved osteoinductive potential and extracellular matrix mineralization (BMP2). Higher proliferation rates observed in MTS assays further support sustained cell viability and growth on Scaffold2.

## Discussion

### HA-mediated reinforcement and degradation

The XRD analysis of the synthesized HA powder revealed that no CaCO₃ or CaO peaks were present, indicating that no residual precursor or secondary phases remained after synthesis and suggesting a complete transformation of calcium carbonate into HA. These findings are consistent with previous reports [48], confirming the successful synthesis of phase-pure HA powder. SEM analysis of the HA powder revealed flaky and elongated particles, which are attributed to the hydrothermal processing conditions. Parameters such as temperature, reaction time, pH, and additives are known to significantly influence HA crystal growth. For example, increasing the reaction temperature (e.g., up to 200 °C) enhances crystallinity, resulting in sharper XRD peaks and potential morphological changes [49]. Moreover, the hydrothermal method is reported to produce defect-free structures with reduced hydroxyl dislocations, which may further affect the crystalline structure and solubility of HA nanoparticles [29, 50]. BET analysis of the synthesized HA powder indicated a specific surface area of 23 m²/g, which falls within the typical range for synthetic HA and is higher than that of bone-derived HA ( ~ 0.1 m^2^/g) [51]. The relatively high surface area and internal porosity are advantageous for bone defect repair, as increased surface area enhances mineral absorption and provides more active sites for biological interaction and degradation. Porous HA exhibits higher absorption rates due to increased interaction with physiological fluids and tissues, which can induce mechanical stress in thin scaffold structures and accelerate osteoclast-mediated resorption [52]. Furthermore, the enhanced porosity promotes bone ingrowth and natural remodeling around the scaffold, facilitating material turnover [53].

Mechanical testing of the scaffolds revealed that the incorporation of HA nanoparticles enhanced both the compressive modulus and strength across all samples, in agreement with previous studies [54, 55]. This improvement is attributed to HA nanoparticle reinforcement, which effectively strengthens the pure PCL matrix. As shown in Table 1, the compressive modulus values of the 3D-printed scaffolds with increasing HA content fall within the typical range for trabecular bone (0.1–0.3 GPa) [55–57]. However, excessive HA content may negatively impact mechanical performance due to particle discontinuities, as observed in the scaffold containing 30 wt.% HA. The scaffold composed of 75 wt.% PCL and 25 wt.% HA exhibited the highest combined compressive modulus and strength and was therefore selected for further investigation.

To evaluate scaffold degradation in PBS, the weight loss of the PCL/HA (25 wt.%) scaffold was monitored over time. Incorporation of HA nanoparticles enhances the polarity and hydrophilicity of polymer-based scaffolds, thereby accelerating degradation. The observed weight loss trend can be attributed to nanoparticle dispersion and capillary water absorption, which facilitate hydrolytic degradation of the polymer matrix through improved water penetration at ester linkages [58, 59]. These results are consistent with previous studies reporting that HA incorporation enhances the biodegradability of PCL-based scaffolds [60]. The changes in pH of the PBS solution were monitored to further assess scaffold degradation. The initial rise in pH is likely due to HA degradation, releasing calcium and phosphate ions, whereas the subsequent pH decrease is mainly caused by acidic byproducts from PCL degradation, particularly from carboxyl-terminal groups. HA particles counteract polymer acidity while promoting degradation, owing to their porous nature, which facilitates water absorption and diffusion [61, 62].

### Apatite formation mechanism

To assess scaffold bioactivity in SBF, pH changes of the solution were monitored over time. The initial rise in pH is likely due to the release of OH⁻ ions from calcium hydroxide in the HA nanoparticles, consistent with previous reports [62]. Ion exchange between H⁺ ions from SBF and Ca²⁺ ions on the scaffold surface may also contribute to this increase [63]. The subsequent decrease in pH is attributed to the consumption of OH⁻ ions during apatite precipitation, suggesting dissolution of ionic species from both PCL and HA nanoparticles in the SBF solution [64]. Changes in calcium and phosphorus ion concentrations were measured to further evaluate apatite formation on the scaffold surface. The observed reduction in calcium and phosphorus concentrations indicates accelerated apatite nucleation and growth. The depletion of these ions from the SBF solution supports active formation of apatite layers. Previous studies have demonstrated that HA nanoparticles enhance apatite crystallization in SBF, thereby improving the bioactivity of PCL-based scaffolds [65]. Immersing scaffolds in SBF is an effective method for evaluating apatite-forming ability. Apatite formation largely depends on the type of bioceramic present; since HA is a major component, surfaces containing HA significantly facilitate apatite deposition. In contrast, pure PCL scaffolds exhibit lower apatite formation due to a lack of inherent bioactivity and potential dissolution of apatite in SBF over time. Surface porosity also plays a critical role in apatite formation, as materials with higher porosity tend to promote greater apatite deposition [66, 67]. The presence of surface functional groups enhances ion exchange between the scaffold and the surrounding solution [68]. Specifically, negatively charged OH⁻ and PO₄³⁻ groups within HA and the presence of Ca²⁺ ions serve as nucleation sites, promoting continuous mineralization and repeated cycles of HA layer formation [69]. These findings are consistent with previous studies, including Afza et al. [70], which demonstrated that precipitation of calcium and phosphorus on PCL/HA scaffold surfaces increased with longer immersion times in SBF. The formation of an apatite layer on the scaffold surface after immersion in SBF confirms the scaffold’s bioactive potential, consistent with previous studies [71, 72]. The increase in the number of interacting peaks suggests a higher degree of mineralization on the scaffold surface, indicating effective interaction of calcium and phosphate ions with the scaffold’s porous structure.

### Zn-induced osteogenic activity

To evaluate scaffold microstructure and nanoparticle distribution, SEM imaging and elemental mapping were performed. The observed pore size of approximately 350 µm exceeds the minimum required for effective cell interaction ( ~ 100 µm) and is generally suitable for vascular growth and new bone regeneration [73, 74]. The uniform distribution of HA nanoparticles within the PCL matrix is consistent with previous studies [75] and suggests effective nanoparticle incorporation, which may enhance both bioactivity and mechanical reinforcement. Additionally, the SEM and elemental mapping results for Scaffold2 confirm successful deposition of the ZIF-8 layer in the present study. These findings align with previous reports [40, 76–78], where in-situ synthesis of ZIF-8 nanoparticles produced spherical particles (50–300 nm) containing C, N, O, and Zn. This supports the potential application of ZIF-8-modified scaffolds for wound healing, as demonstrated in the study by Maghsoudi et al. [78].

To evaluate the release behavior of Zn²⁺ ions, the scaffold was monitored over time in PBS. The prolonged and regulated release of Zn²⁺ ions suggest a synergistic interplay between diffusion-controlled transport and gradual biodegradation of the scaffold [79]. The persistent presence of Zn²⁺ not only promotes osteogenic differentiation but may also confer antibacterial functionality, enhancing the multifunctionality of the scaffold [79]. When combined with the apatite-forming potential of hydroxyapatite and the mechanical stability of the PCL matrix, the controlled Zn²⁺ release contributes to a scaffold capable of simultaneous bone-regenerative and protective functions [79]. Although the observed Zn²⁺ concentrations remain within safe biological limits (~3–100 µM) [80], considerations regarding long-term stability and potential zinc accumulation remain critical. Extended in vitro and in vivo studies are warranted to fully assess scaffold degradation and the physiological impact of prolonged Zn²⁺ exposure.

Fluorescent staining with DAPI and phalloidin was performed to assess early cell adhesion and morphology on the scaffolds. The 3-day adhesion results indicate that both Scaffold1 and Scaffold2 provide favorable surfaces for MG-63 cell attachment. Scaffold2 appears to enhance cell-surface interactions, likely due to surface modifications that improve hydrophilicity and bioactivity. The observed prolonged viability and osteogenic marker expression suggest that the scaffold design effectively supports osteogenic differentiation. While the 3-day data provide valuable insight into early cell-scaffold interactions, longer-term adhesion and proliferation assessments could further clarify sustained effectiveness.

MTS assays were conducted to evaluate cell viability and proliferation over time. The observed enhanced adhesion and proliferation on Scaffold2 suggest that surface modifications, such as ZIF-8 functionalization, improve cell-scaffold interactions and bioactivity [9, 40, 44]. The exponential increase in cell growth confirms that both scaffolds provide a supportive microenvironment for MG-63 cells, maintaining viability and function. These results reinforce the scaffolds’ safety and biocompatibility, highlighting their suitability for bone tissue engineering applications.

Real-time PCR analysis was performed to assess osteogenic differentiation at the molecular level. The upregulation of ALP and BMP2 in Scaffold2 can be attributed to the controlled release of Zn²⁺ ions from ZIF-8, which activate osteogenic signaling pathways such as BMP/SMAD and Wnt/β-catenin [40]. Zn^2+^ is known to enhance the activity of transcription factors like Runx2, promoting osteogenic differentiation [9, 40, 44]. The increased BMP2 expression suggests that ZIF-8 enhances scaffold bioactivity, providing an osteoconductive environment that stimulates cellular differentiation and matrix deposition. The higher proliferation rates observed in MTS assays support the conclusion that ZIF-8 not only promotes osteogenic differentiation but also maintains a favorable environment for sustained cell viability. These findings confirm that ZIF-8 modification significantly improves the osteogenic performance of PCL/HA scaffolds, consistent with previous studies showing that surface coatings on scaffolds enhance osteoblast attachment and activity [79]. Future studies should investigate the long-term impact of Zn²⁺ release on bone remodeling and scaffold degradation in vivo.

### Synergistic scaffold design

The compressive properties, together with the cellular responses, indicate the potential applicability of the fabricated scaffold in this study for trabecular bone regeneration. Although the compressive modulus of the fabricated scaffold is comparable to that of native trabecular bone, the measured compressive strength ( ~ 17 MPa) lies toward the lower-to-mid range reported for cancellous bone. It is important to interpret this value in the context of the intended application. The scaffold was primarily designed to mimic trabecular bone architecture rather than cortical bone, and therefore is not intended for high load-bearing applications. Instead, it is more suitable for cancellous bone defects or non-critical load-bearing regions, where mechanical support is required temporarily until new bone formation occurs. Furthermore, the relatively moderate compressive strength can be attributed to the high porosity of the scaffold, which was intentionally introduced to enhance cell infiltration, nutrient diffusion, vascularization, and overall biological performance. It is well established that increasing porosity improves biological functionality but inevitably reduces absolute mechanical strength due to decreased solid fraction and load transfer pathways. Therefore, the mechanical performance observed in this study reflects a deliberate balance between structural integrity and biological efficacy. Within this framework, the obtained compressive strength remains within the reported range for trabecular bone and is considered adequate for the proposed regenerative application [81].

While the present study provides valuable insights into the fabrication and bioactivity of PCL/HA@ZIF-8 scaffolds, several limitations should be acknowledged. The in vitro experiments, though informative, cannot fully replicate the complex physiological environment, and thus in vivo studies are necessary to confirm scaffold performance under more realistic conditions. Additionally, investigating alternative functionalization strategies, long-term degradation behavior, and sustained ion release could provide a deeper understanding of scaffold efficacy. Scaling up the production of such nanocomposite scaffolds for clinical applications may encounter challenges, including reproducibility, cost, regulatory compliance, and safety considerations. The complexity of the manufacturing process and the need for specialized expertise further emphasize the importance of a coordinated approach. Addressing these challenges will require technological innovation, stringent quality control, and close collaboration among researchers, manufacturers, and regulatory bodies. Despite these hurdles, overcoming them has the potential to translate these scaffolds into effective clinical solutions for bone tissue engineering and regenerative medicine.

## Conclusion

This study successfully developed a multifunctional 3D-printed PCL/HA scaffold surface-modified with in-situ ZIF-8 to enhance bone regeneration. The optimal 25 wt.% HA composition provided mechanical properties (compressive modulus ~0.36 GPa and strength ~17 MPa) within the range reported for human trabecular bone, alongside controlled biodegradation ( ~ 15% weight loss over 28 days) and robust bioactivity demonstrated by progressive apatite formation in SBF. ZIF-8 functionalization introduced sustained, non-burst Zn²⁺ release (0.18–1.66 ppm over 28 days) within safe limits, significantly improving in vitro cellular performance. MG-63 cells exhibited enhanced adhesion, proliferation, and osteogenic differentiation on modified scaffolds, with 2.1-fold upregulation of ALP and 2.5-fold upregulation of BMP2 expression compared to unmodified controls. These findings highlight the synergistic benefits of HA reinforcement for osteoconductivity and ZIF-8-mediated Zn²⁺ delivery for osteoinductivity, offering a promising platform for cancellous bone defects or non-load-bearing applications. While in vitro results confirm cytocompatibility and enhanced regenerative potential, future in vivo studies are essential to evaluate long-term integration, degradation kinetics, and clinical efficacy in relevant defect models. Overall, this approach advances bone tissue engineering by combining precise 3D architecture with bioactive ion release, paving the way for more effective scaffold-based therapies.

## Data Availability

The data presented in this study are available on request from the corresponding author.
